# Historical and regional particularities in the prevalence of traumatic events and posttraumatic stress disorder in East and West Germany

**DOI:** 10.1186/s12889-023-16534-6

**Published:** 2023-08-23

**Authors:** Christoph Kasinger, Ann-Christin Schulz, Christine Ulke, Andreas Maercker, Manfred Beutel, Elmar Brähler

**Affiliations:** 1 Department of Psychosomatic Medicine and Psychotherapy, University Medical Center of the Johannes Gutenberg University Mainz, Heidestraße 146, 60385 Frankfurt am Main, Germany; 2 Department of Psychiatry and Psychotherapy, University of Leipzig Medical Center, Leipzig, Germany; 3 Department of Psychology, Division Psychopathology and Clinical Intervention, University of Zurich, Zuerich, Switzerland

**Keywords:** Trauma, PTSD, Traumatic events, East/West Germany, Prevalence

## Abstract

**Background:**

Epidemiological research on the prevalence of traumatic events and PTSD has shown that there are significant differences between countries, due to their different history and socialization processes. In the case of Germany, this is particularly relevant. Germany was divided into two states from 1949 to 1990. This study examines the prevalence of traumatic events and PTSD in the formerly divided East and West Germany.

**Methods:**

For the prevalence of traumatic events, we used data from four representative surveys (years 2005, 2007, 2008, and 2016) with a total of *N* = 9,200 respondents. For the analyses of PTSD prevalence, we used data from three representative surveys (years 2005, 2007, 2008) with a total of *N* = 6676 respondents. We compared different birth cohorts, persons living in the former West vs. East Germany, and the application of different diagnostic criteria using a chi-square test.

**Results:**

The overall one-month prevalence rate for PTSD was 3.4% (3.0% for men and 3.8% for women). We found significant differences in the occurrence of traumatic events between genders, different age cohorts as well as between people who live in East and West Germany. Significant differences in the prevalence of PTSD can only be observed for different age cohorts. Most of the age effects are due to traumatic events related to World War II (WWII). Prevalence rates for PTSD were higher when the diagnostic criterions of the DSM-V were applied compared to the criterions of the DSM-IV.

**Conclusions:**

Our data suggests that socio-political factors may need to be considered when accounting for differences in occurrence rates of traumatic events, but not for prevalence rates of PTSD, between East and West Germany. People who have experienced WW II have a higher risk of suffering from PTSD. Future epidemiological trauma research should take historical and regional peculiarities of countries into account.

## Background

Traumatic events (TEs) are emotionally disturbing and often involve a threat to a person's life, physical well-being, or psychological integrity and are typically perceived as overwhelming, frightening, or shocking, and may lead to a sense of helplessness or loss of control. Experiencing a traumatic event (TE) can cause severe stress and while most people recover from it without mental impairment [1] others develop a post-traumatic stress disorder (PTSD). The Post-traumatic stress disorder (PTSD) is a mental disorder that, according to the ICD-11 definition, arises as the affected person’s reaction to a stressful event or situation of exceptional threat or catastrophic proportions (traumatic event).This includes life-threatening illness or injury, war, terrorist attacks, or abuse [2].

In the 4^th^ edition of diagnostic and statistical manual of mental disorders of the American Psychiatric Association (DSM-IV) [3], Criterion A2 stated that the person’s response to the traumatic event involved intense fear, helplessness, or horror. This criterion was removed in the DSM-5 [4]. The emphasis shifted from the person's emotional response to the objective nature of the traumatic event itself. These changes in Criterion A were made to acknowledge that individual subjective responses to traumatic events can vary. This raises the question, if the change in the diagnostic criterions (removal of criteria A2) impacts the prevalence rates of PTSD. Therefore, the results of the prevalence rates of PTSD are presented separately for the criterion A according to the DSM-IV and for criterion A according to the DSM-V.

Due to its substantial psychological impairment, chronic course and high comorbidity with other mental disorders, like depression, anxiety disorders and substance abuse [5, 6] PTSD is an highly relevant disorder in the field of psychiatry and clinical psychology. Even if people do not develop PTSD, the experience of a traumatic event increases the likelihood of hospitalalizationand increases the demand for psychosocial care [7]. The first step to tackle the clinical and economic challenges TEs and PTSDs cause is to determine the prevalence rates in the general population.

Epidemiological studies on the prevalence of PTSD and TEs draw an inconsistent picture regarding different countries. The data availability for period prevalence of TEs are scarce. According to Frazier et al., [8] the 2-month prevalence of traumatic events for undergraduate students is 21%. Lifetime prevalence for TEs in a worldwide sample of the WHO is 70.4% [9]. In a Dutch population the prevalence of TEs ranges between 43.8% [10] and 80.7% [11]. Among people living in Germany lifetime prevalence rates for TEs range between 24% [12] and 41% [13]. The prevalence rates for PTSD draw an inconsistent picture as well. A meta-analyses by Steel et al. [14] found point-prevalence rates for PTSD in populations in post conflict countries worldwide around 30.6% with ranges between 0 to 99%. In a study by Karam et al. [15] using the World Mental Health Surveys data, the 12-months prevalence rates for PTSD in the general population ranged between 3.8% in Northern Ireland and 2.5% in the United States to 0.2% in Beijing and Shanghai and 0.3% in Mexico and Colombia. A review by Schein et al. [16] found point prevalence rates for PTSD in the US among civilians ranges from 8% to 56.7%. Regarding 1-year prevalence rates for PTSD they state that among civilians the rate ranges from 2.3% to 9.1%. The lifetime prevalence among civilians ranges from 3.4% to 26.9% and 7.7% to 17% for the military population. Another study by Burri and Maercker [17] investigated the 12-months prevalence rates of PTSD in 11 European countries and found prevalence rates ranging from 0.38% (Belgium) up to 6.67% (Croatia). While it remains unclear precisely what causes these differences, the prevalence of PTSD also depends on an individual's socio-political and historical context. For example, Burri and Maercker [17] identified exposure to war related experiences as one possible explanation for the differences in prevalence rates.

One-month prevalence rates for PTSD in Germany range between 1.4% [18], 1.5% [19], 2.3% [20] up to 2.9% [12].

Considering that the socio-political and historical environment also influences PTSD and TEs, it is necessary to consider the case of Germany, with its history of two different socio-political environments when screening for TEs and PTSD. After World War II Germany was assigned into four different occupation zones, which were administered by the four main allies. In 1949 the zones occupied by the Western Allies formed the liberal and capitalistic Federal Republic of Germany (FRG). The occupation zone of the Soviet Union was transformed into the socialistic German Democratic Republic (GDR). Both states had very different legal, economic, and social frameworks that affected people living in these states in different ways. For example, the two states had a different attitude toward corporal punishment, with the GDR banning it earlier than the FRG [21]. The two states also differed in terms of women’s rights and gender equality, as women in the GDR were granted the same rights as men and abortion were ruled legal in 1972 as it was seen as one necessary factor in achieving gender equality [22]. Since gender inequality is a risk factor for sexual and physical violence [23, 24] it is possible that prevalence rates of these TE were smaller in the GDR due to the higher level of gender equality. On the other hand, unlike in the FRG people in the GDR were subjected to the terror of the Stasi, a powerful secret police force, that tortured, exerted emotional, financial, and social repression, and created an atmosphere of mistrust [25] thataffected the mental health of the victims [26].

To our knowledge this is the first study investigating if there are different prevalence rates for PTSD and TEs in East and West Germany.

## Methods

### Participants

Participants were surveyed as part of  German representative surveys in 2005 (REP 12), 2007 (REP 14), 2008 (REP 15a) and 2016 (REP 24). These are combined to analyze the lifetime prevalence of traumatic events. Only the first three (2005, 2007, 2008) are used to establish the one-month-prevalence of PTSD as the corresponding items did not appear in REP 24. Participants have been chosen and data has been collected by a demographic consulting company (USUMA, Berlin, Germany) using a random-route procedure and random selection of a member of the identified households to assemble a representative sample. After providing informed consent, a total of 9,200 participants aged 18 to 93 (N_female_ = 4,987, M_age_ = 52.5, SD_age_ = 18.4) answered the relevant questions, of which 6,693 participants (N_female_ = 3,635, M_age_ = 53.8, SD_age_ = 18.5) took part in REPs 12, 14 and 15a, and 2,507 (N_female_ = 1,352, M_age_ = 49.21, SD_age_ = 17.29) in REP 24. Further sociodemographic information can be seen in Table 1. Ethical approval was granted by the institutional ethics review board of the University of Leipzig.

**Table 1:** Sample description for relevant socio-demographic variables

	Total (*N* = 9,200)	Sample survey years 2005, 2007, 2008 (*N* = 6,693)	Sample survey year 2016 (*N* = 2,507)
	N (%); M ± SD	N (%); M ± SD	N (%); M ± SD
Gender (Female)	4,987 (54.2)	3,635 (54.3)	1,352 (53.9)
Age	52.5 (18.4)	53.8 (18.5)	49.21 (17.9)
Married (Yes)	5,498 (59.8)	4,092 (61.1)	1,406 (56.1)
Net Household income (≥ 2000 €/month)	2,315 (25.2)	1,408 (21.0)	907 (36.2)
Cohorts			
< = 1946	3,397 (36.9)	2,542 (38.0)	855 (34.1)
1947–1968	3,182 (34.6)	2,201 (32.9)	981 (39.1)
> = 1969	2,621 (28.5)	1,950 (29.1)	671 (26.8)
Unemployed (Yes)	526 (5.7)	329 (4.9)	197 (7.9)
East-/West German (West German)	7,290 (79.2)	5,286 (79.0)	2,004 (79.9)

## Measures

To determine whether participants live in West or East Germany, they were asked where they currently live. Participants living in Berlin were assigned to East or West Berlin.

### List of traumatic events

The trauma list of the Munich Composite International Diagnostic Interview [27] consists of eight potentially traumatizing events (e.g. “you were the victim of rape”, “… of a natural disaster”; “you had horrible experiences during war service”), and a question regarding the experience of “another terrible event or a catastrophe”. Two war-related items were added (“You were bombed”, “You were driven out of your homeland”). Participants responded in a dichotomous format (yes / no). Additionally, participants answered an item regarding the experience of intense fear and helplessness according to the DSM-IV A2 criterion of PTSD. If participants had experienced more than one potential traumatic event, they were asked to identify the subjectively most painful event to which the following questions were related.

### Modified Posttraumatic Diagnostic Scale (German Version)

The German version of the Posttraumatic Diagnostic Scale [28, 29] was used to screen for symptoms of PTSD. However, the items corresponding to criteria B3, B4, C2, C3 and D2–D4 of the Diagnostic and Statistical Manual of Mental Disorder, version IV (DSM-IV) were omitted as Breslau et al. [30] have found these items have low sensitivity and specificity and therefore be of low diagnostic value. The resulting scale includes 11 items regarding intrusions, avoidance and hyperarousal that were answered on a 4-point Likert scale ranging from 0 (“not at all) to 3 (“several times a week / almost always”). Items marked 0 or 1 (“once a week or less”) were counted as negative and those marked 2 (“2–4 times a week / half of the time”) or 3 were counted as positive. Additionally, the duration of the symptoms according to criterion E of DSM-IV has been screened. With a sensitivity of 80%, a specificity of 97%, a positive predictive value of 71% and a negative predictive value of 98% and an internal consistency with Cronbach’s alpha of α = 0.94 [30], the scale is well suited for screening for PTSD-symptoms.

To diagnose PTSD according to DSM-IV, items relating to criteria A1, A2 and four out of seven Symptoms relating to the B-criteria had to be marked as positive. Additionally, subclinical syndromes have been analyzed by specifying partial PTSD which has been diagnosed when at least two symptoms of criteria B to D are marked positive while the F-criterion is absent.

### Statistical analysis

All statistical analyses were run in the SPSS version 26. Descriptive statistics were calculated separately for gender, place of residence, and three age cohorts. Three age cohorts were calculated for those born before 1947, born between 1948 and 1968, and after 1969. The first age cohort covers those participants who have been directly impacted by WWII. The second age cohort are those who were born in post war Germany and the third age cohort covers those participants who were not influenced by war related factors anymore. Additionally, chi-square tests were conducted to analyze possible differences between gender, former place of residence and age cohorts. One-month prevalence rates of full and partial PTSD were calculated for each group. To reflect recent developments in traumatic researchthese rates were analyzed for criteria A1 and A2 and for criterion A1 only.

## Results

Sociodemographic characteristics for the different samples are reported in Table 1.

### Prevalence of TEs in the total sample and stratified by gender, place of residence and birth cohort

The prevalence of lifetime traumatic events is shown in Tables 2 and 3. The prevalence of at least one traumatic life event in the total sample was 27.2% (*N* = 1,818). There are no significant gender differences. However, women reported rape and experienced sexual child abuse more often, whereas men experienced physical violence, serious accidents, imprisonment or hostage-taking more frequently.

**Table 2:** Prevalence of TEs between Gender and East/West Germany

	Total *n* (%)	Male – West *n* (%)	Male – East *n* (%)	X^2^	Female– West *n* (%)	Female – East *n* (%)	X^2^	Total – Male *n* (%)	Total – Female *n* (%)	X^2^	Total – West *n* (%)	Total – East *n* (%)	X^2^
War Deployment (*N* = 9,139)	661 (7.2)	249 (7.5)	53 (6.1)	2.14	287 (7.3)	72 (7.1)	0.40	302 (7.2)	359 (7.3)	0.10	536 (7.4)	125 (6.6)	1.30
Bombed Out (*N* = 6,660)	483 (7.3)	182 (7.5)	31 (4.8)	6.02 *	217 (7.6)	53 (7.1)	0.21	213 (7.0)	270 (7.5)	0.72	399 (7.6)	84 (6.0)	3.94*
Physical Violence (*N* = 9,141)	443 (4.8)	230 (6.9)	40 (4.6)	6.42 *	145 (3.7)	28 (2.8)	2.04	270 (6.4)	173 (3.5)	43.01**	375 (5.2)	68 (3.6)	8.02 **
Rape (*N* = 9,137)	65 (1.4)	13 (0.4)	2 (0.2)	0.47	89 (2.3)	23 (2.3)	0.00	15 (0.4)	112 (2.3)	59.95**	102 (1.4)	25 (1.3)	0.07
Sexual Child Abuse (*N* = 9,135)	144 (1.6)	26 (0.8)	5 (0.6)	0.42	92 (2.3)	21 (2.1)	0.25	31 (0.7)	113 (2.3)	34.76**	118 (1.6)	26 (1.4)	0.59
Displacement (*N* = 6,664)	494 (7.4)	161 (6.7)	54 (8.3)	2.13	191 (6.7)	88 (11.8)	21.47**	215 (7.0)	279 (7.7)	1.27	352 (6.7)	142 (10.2)	19.59**
Natural Disaster (*N* = 9,135)	137 (1.5)	49 (1.5)	22 (2.5)	4.45 *	52 (1.3)	14 (1.4)	0.02	71 (1.7)	66 (1.3)	2.01	101 (1.4)	36 (1.9)	2.65
Severe Accident (*N* = 9,139)	527 (5.7)	257 (7.8)	59 (6.8)	0.99	168 (4.3)	43 (4.2)	0.00	316 (7.6)	211 (4.3)	45.23**	425 (5.9)	102 (5.4)	0.58
Captivity (*N* = 9,143)	128 (1.4)	76 (2.3)	24 (2.7)	0.61	19 (0.5)	9 (0.9)	2.34	100 (2.4)	28 (0.6)	54.66**	95 (1.3)	33 (1.7)	2.07
Life Threatening Disease (*N* = 9,142)	400 (4.3)	149 (4.5)	42 (4.8)	0.15	157 (4.0)	52 (5.1)	2.64	191 (4.6)	209 (4.2)	0.63	306 (4.2)	94 (5.0)	2.07
Witness of a TE (*N* = 9,137)	936 (10.2)	349 (10.5)	71 (8.1)	4.34 *	434 (11.0)	82 (8.1)	7.41**	420 (10.0)	516 (10.4)	0.39	783 (10.8)	153 (8.1)	11.75**
Other TE (*N* = 9,074)	443 (4.9)	134 (4.1)	31 (3.6)	0.46	209 (5.3)	69 (6.8)	3.21	165 (4.0)	278 (5.6)	13.56**	343 (4.8)	100 (5.3)	0.96
At Least One TE (*N* = 6,676)	1.818 (27.2)	673 (27.9)	175 (26.8)	0.29	747 (26.1)	223 (29.7)	3.81	848 (27.6)	970 (26.9)	0.48	1.420 (26.9)	398 (28.3)	1.12

*Note*. Significant levels: *p < .05, **p < .01

**Table 3:** Prevalence of TEs regarding age cohorts and East/West Germany

	< = 1946			1947–1968			> = 1969			Total
	West *n* (%)	East *n* (%)	X^2^	West *n* (%)	East *n* (%)	X^2^	West *n* (%)	East *n* (%)	X^2^	X^2^ (df = 2)
War Deployment (*N* = 9,139)	473 (18.3)	116 (14.6)	5.79*	26 (1.0)	4 (0.7)	0.73	37 (1.7)	5 (1.1)	1.18	831.11**
Bombed Out (*N* = 6,660)	376 (16.2)	82 (11.7)	8.40**	13 (0.7)	1 (0.2)	1.40	10 (0.8)	1 (0.4)	0.61	512.39**
Physical Violence (*N* = 9,141)	179 (6.9)	38 (4.8)	4.72*	91 (3.6)	10 (1.6)	6.10*	105 (5.0)	20 (4.2)	0.48	36.83**
Rape (*N* = 9,137)	30 (1.2)	8 (1.0)	0.14	29 (1.1)	8 (1.3)	0.11	43 (2.0)	9 (1.9)	0.03	9.95**
Sexual Child Abuse (*N* = 9,135)	30 (1.2)	4 (0.5)	2.62	40 (1.6)	7 (1.1)	0.64	48 (2.3)	15 (3.1)	1.29	19.24**
Displacement (*N* = 6,664)	302 (13.0)	138 (19.7)	19.38**	32 (1.8)	2 (0.5)	4.16*	18 (1.5)	2 (0.7)	0.92	409.44**
Natural Disaster (*N* = 9,135)	37 (1.4)	13 (1.6)	0.16	36 (1.4)	8 (1.3)	0.05	28 (1.3)	15 (3.2)	7.98**	0.69
Severe Accident (*N* = 9,139)	170 (6.6)	39 (4.9)	2.92	158 (6.2)	38 (6.2)	0.00	97 (4.6)	25 (5.2)	0.39	7.69*
Captivity (*N* = 9,143)	72 (2.8)	29 (3.6)	1.53	8 (0.3)	2 (0.3)	0.00	15 (0.7)	2 (0.4)	0.50	99.10**
Life Threatening Disease (*N* = 9,142)	168 (6.5)	60 (7.5)	1.05	106 (4.2)	30 (4.9)	0.59	32 (1.5)	4 (0.8)	1.26	100.92**
Witness of a TE (*N* = 9,137)	393 (15.2)	78 (9.8)	14.93**	222 (8.7)	36 (5.9)	5.38*	168 (7.9)	39 (8.2)	0.04	79.96**
Other TE (*N* = 9,074)	159 (6.2)	52 (6.5)	0.13	103 (4.1)	32 (5.2)	1.52	81 (3.9)	16 (3.4)	0.22	22.92**
At Least One TE (*ss* = 6,676)	974 (41.8)	296 (42.0)	0.01	285 (16.4)	62 (14.5)	0.97	161 (13.4)	40 (14.8)	0.37	603.78**

*Note*. Significant levels: *p < .05, **p < .01

Birth cohort had a significant impact on the prevalence of traumatic life events. Older participants experienced more war-related traumatic events like war deployment, being bombed out or being taken as a hostage and physical violence. Being displaced, experiencing a life-threatening disease and being a witness of a traumatic event were also more frequently reported by older participants. On the contrary rape and child abuse were more often reported in younger cohorts.

Physical violence and being a witness of a traumatic event were more frequently reported in participants in West Germany, while people in East Germany more often reported having been displaced from their home country. Women from East Germany were much more likely to report being expelled from their home country, whereas women from West Germany witnessed a traumatic event more often. Interestingly, except for experiencing a natural disaster, significant differences in TE frequencies between East and West Germany can only be observed for people born before 1969.

### Prevalence PTSD in general, between gender, place of residence and different birth cohorts

Results for the distribution of the prevalence of PTSD and partial PTSD are shown in Table 4. When applying the A1-Criterion only, as DSM-5 states, the prevalence of PTSD in the total sample was 3.4%. Prevalence rates for the total sample decrease to 2.7% when adding the A2-Criterion. The prevalence of partial PTSD in this sample was 5.3% for the A1-Criterion and 3.9% when adding the A2-Criterion respectively.

**Table 4:** Prevalence of PTSD in general, between sexes, former place of residence and different birth cohorts

	Total *n* (%)	Male *n* (%)	Female *n* (%)	X^2^	West *n* (%)	East *n* (%)	X^2^	< = 1946 *n* (%)	1947–1968 *n* (%)	> = 1969 *n* (%)	X^2^
1. PTSD (A1)	228 (3.4)	91 (3.0)	137 (3.8)	3.47	184 (3.5)	44 (3.1)	0.43	135 (4.5)	59 (2.7)	34 (2.3)	18.55**
2. PTSD (A1 + A2)	180 (2.7)	66 (2.2)	114 (3.2)	6.43*	143 (2.7)	37 (2.6)	0.03	96 (3.2)	54 (2.5)	30 (2.0)	5.37
3.Partial PTSD (A1)	352 (5.3)	170 (5.5)	182 (5.0)	0.82	278 (5.3)	74 (5.3)	0.00	225 (7.4)	81 (3.7)	46 (3.1)	51.92**
4.Partial PTSD (A1 + A2)	260 (3.9)	109 (3.6)	151 (4.2)	1.77	201 (3.8)	59 (4.2)	0.45	141 (4.6)	83 (3.8)	36 (2.4)	12.99**

*Note*. Significant levels: *p < .05, **p < .01

Significant sex differences can only be found for full PTSD with A1- and A2-Criterion, with women reporting higher rates of PTSD. No significant differences in the prevalence of PTSD or partial PTSD for East and West Germans was found. Birth cohort had a significant influence again, as older participants showed higher prevalence rates of PTSD (A1) and partial PTSD. Interestingly, this significant difference did not occur when applying the A1- and A2-Criterion for full PTSD.

### PTSD – Conditional probability of developing PTSD

The average conditional probability of developing a PTSD in the presence of a traumatic life event was 12.5% in this representative sample. Traumatic life events with the greatest probability of developing PTSD were child abuse (41.7%), rape (40.9%) and life-threatening disease (26.5%). Regarding partial PTSD a serious accident (30.4%), being taken as a hostage (28.5%), being a witness of a traumatic life event (27.2) and life -threatening disease (25.9%) have been identified as traumatic life events with the greatest probability of developing a partial PTSD. Again, applying the A1 vs. the A1- and A2-Criterion changed some of the rates for conditional probability of developing a PTSD or partial PTSD. Table 5 shows the results for the conditional probability of developing a PTSD in dependence of the different traumatic events.

**Table 5:** Conditional Occurrence of PTSD and partial PTSD

	Subjectively Worst Traumatic Experience	Conditional Occurrence of PTSD		Conditional Occurrence of partial PTSD	
		A1	A1 + A2	A1	A1 + A2
	*n* (%)	*n* (%)	*n* (%)	*n* (%)	*n* (%)
War Deployment	192 (14.1)	29 (15.1)	24 (12.5)	34 (17.7)	23 (12.0)
Bombed Out	135 (9.9)	11 (8.1)	4 (3.0)	16 (11.9)	6 (4.4)
Physical Violence	49 (3.6)	9 (18.4)	6 (12.2)	11 (22.4)	8 (16.3)
Rape	22 (1.6)	9 (40.9)	8 (36.4)	3 (13.6)	2 (9.1)
Sexual Child Abuse	36 (2.6)	15 (41.7)	14 (38.9)	6 (16.7)	6 (16.7)
Displacement	172 (12.6)	14 (8.1)	8 (4.7)	33 (19.2)	16 (9.3)
Natural Disaster	13 (1.0)	2 (15.4)	2 (15.4)	1 (7.7)	1 (7.7)
Severe Accident	168 (12.4)	20 (11.9)	17 (10.1)	51 (30.4)	43 (25.6)
Captivity	21 (1.5)	2 (9.5)	0 (0.0)	6 (28.6)	4 (19.0)
Life Threatening Disease	170 (12.5)	45 (26.5)	25 (14.7)	44 (25.9)	28 (16.5)
Witness of a TE	224 (16.5)	27 (12.1)	16 (7.1)	61 (27.2)	38 (17.0)
Other TE	158 (11.6)	32 (20.3)	24 (15.2)	40 (25.3)	34 (21.5)

## Discussion

One main finding is that around 27.2% of the participants have experienced at least one TE. While 41.9% of the participants born before the year 1947 report having experienced at least one TE, the prevalence rate of TEs for younger birth cohorts ranges between 13.6 – 16.0%. The large significant difference between the different birth cohorts can be explained mainly by WWII related TEs like war deployment, being bombed and experiences of displacement. Furthermore, we found prevalence rates for PTSD in Germany around 3.4% when applying the A-criterion of the DSM-V and a prevalence rate of around 2.7% when applying the A-criterion of the DSM-IV (A2-Criterion is included). Interestingly, the significant age difference disappears when the A2-Criterion is included. This indicates that the age effect on the prevalence rate of PTSD is sensitive to the diagnostic criteria. Since there are uncertainties regarding the age effect of PTSDs these results suggest that this may also be due to different diagnostic criteria applied. Considering the removal of the A2-Criterionin the DSM-V we expect more older people be diagnosed with PTSD in the German population. These findings are contrary to other research results who did not find age differences in the prevalence of PTSD [31] or found higher prevalence rates in young adults [32]. These different results are likely to be explained by the different countries where the studies were investigated. For Germany the older population, mostly civilians at that time, was highly exposed to war related TEs, while people in other countries like Swiss or the United States were not directly affected to such an extent. Other investigations found a strong influence of war related events on the prevalence rate of PTSD [16, 33] as well as evidence for a higher prevalence rate of PTSD for populations in post conflict countries [14].

Even though war related traumatic experiences may not cause actual PTSD symptoms, they increase the risk of developing a PTSD after multiple traumatic experiences [5]. In addition, the probability of experiencing a TE, like suffering from a life-threatening disease, experiencing a severe accident or a natural disaster, increases with age. These results indicate that even after 60 years war-related traumatic experiences continue to negatively affect psychological well-being and mental health.

There was no difference in the overall prevalence rate of PTSD between East and West Germans. Future research should aim to validate these findings before investigating and reporting the prevalence rates of PTSD for Germany collectively. However, there were differences in the prevalence rates of the different TEs. West Germans born before 1947 experienced being bombed out more often than East Germans. This is because of the higher population- and urban density in West Germany, with a lot of war related industrial infrastructure at that time. As a result, West Germany was bombed more heavily than the East German country parts [34]. On the other hand, people born in East Germany before 1947 were more likely to experience displacement, accounting for nearly 20% of the East German sample.. From a clinical perspective this is highly relevant as displaced people and their offspring are more likely to suffer from mental disorders like depression and anxiety than those who have not been displaced [35]. Again, historical events during WWII might be responsible for the difference, since many people fled from former German territories in East Europe to nearby German territories, that become part of East Germany [36]. Interestingly people in West Germany born before 1968 report more experience of physical violence than the people in East Germany. A possible explanation for this difference is the different societal context and different legal frameworks in the former GDR and the former FRG. In the former GDR corporal punishment was banned from its inception in 1949 [37], while in the FRG it was still allowed until 1973 (in Bavaria even until 1983) [21]. The changed law regarding corporal punishment in the FRG might explain, why there was no difference for people born after 1968. This hypothesis is supported by other research, which found a sharp decline in corporal punishment in Sweden after it changed its laws regarding corporal punishment as an educational measure in 1979 [38]. Although differences in specific TEs can be observed between East and West Germans, there was no difference in the overall prevalence of experiencing at least one TE between the two groups. Since TEs are the basis for the development of PTSD, and East and West Germans report TEs at the same prevalence, we assume that this is the reason why there were no differences in the prevalence of PTSD.Regarding the conditional probability of developing PTSD our findings replicate previous findings thatman-made-disasters are associated with a higher conditional probability of developing PTSD [39]. Comparing our prevalence rate of 3.4% with the prevalence rate of people diagnosed with a PTSD and are in treatment (0.7% in the year 2017) [40], our results indicate that PTSD is highly underdiagnosed in the German health care system. This has important implications for patient care and call for improvements in diagnostic accuracy. To ensure more accurate diagnoses, healthcare providers should consider implementing comprehensive diagnostic assessments specifically designed for PTSD to enhance the accuracy of PTSD diagnoses and ensure that individuals receive appropriate treatment and support.

Addressing the issue of underdiagnosis requires actions at both the clinical and political levels. Healthcare providers should receive adequate training and education on recognizing and assessing PTSD symptoms. This training can help improve their diagnostic skills and increase awareness about PTSD among healthcare professionals. A special emphasize should lay on older people, since the prevalence-treatment gap is the highest within older patients [40].

On a political level, it is crucial to allocate resources and funding to mental health services not only in Germany, but particularly in areas affected by war or conflict. This includes providing support for specialized PTSD clinics, increasing the availability of mental health professionals, and implementing public awareness campaigns to reduce stigma surrounding mental health issues. Ultimately, by addressing the underdiagnosis of PTSD and implementing more accurate diagnostic procedures, we can ensure that individuals affected by war trauma receive timely and appropriate care, leading to improved outcomes and a better quality of life for these individuals and their communities.

### Limitations

One limitation of the current work is that PTSD and TEs were investigated using questionnaires with non-clinical interviewers. This might have led to biased declarations of the participants. Another methodological limitation is that surveys that ask about events that occurred a long time ago may be subject to memory distortion effects. Unfortunately, we were not able to identify participants who moved from East to West Germany and vice versa. From 1991 to 2018 alone, about 3.8 million people moved from East Germany to West Germany [41]. With about 66.8 million people [42] living in West Germany, the rate of internal migrants is about 5.68%.Since people are not taken into account who moved there and back, the real rate of internal migrants is propably smaller. Still the relatively high number of people from East Germany, who are now living in West Germany might distort the allocation to East or West Germany in our research.

## Conclusion

Our study findings reveal important insights into the implications for the future, especially in war-related areas worldwide.

Firstly, we observed that individuals of higher age in Germany are more susceptible to experiencing PTSD and traumatic events, predominantly due to their exposure to war-related experiences. This finding highlights the enduring impact of historical events, such as wars, on mental health outcomes. In addition, our results show that most people who are suffering from PTSD do not get adequate treatment in Germany and that PTSD is likely to be underdiagnosed.

Furthermore, while we did not find significant differences in the prevalence of PTSD between individuals who lived in the former German Democratic Republic (GDR) and the former Federal Republic of Germany (FRG), we did identify differences in the prevalence of traumatic events. These results indicate that historical experiences, particularly those related to war and collective trauma, can significantly influence the prevalence rates of both PTSD and traumatic events within a population.

The impact of historical events on mental health outcomes is not limited to war-related experiences; it can also extend to other collective traumatic events, such as famines and natural disasters. Therefore, future research should expand its focus to other regions and populations worldwide that have been exposed to significant historical traumas.

Apart from historical particularities our results reveal a significant impact on the prevalence of PTSD depending on the diagnostic criteria applied.

In conclusion, our study sheds light on the enduring influence of historical experiences on the prevalence of PTSD and traumatic events. As researchers and mental health practitioners, it is crucial to consider the historical context and regional specificities when designing interventions and support systems for populations affected by collective trauma, especially in war-related areas across the world.

## Data Availability

Data and materials are available upon reasonable request from the corresponding author.

## Abbreviations

PTSD: Posttraumatic Stress Disorder
TE: Traumatic Event
GDR: German Democratic Republic
FRG: Federal Republic of Germany
WW II: World War II
